# Synthesis, function, and therapeutic potential of glycosphingolipids

**DOI:** 10.3389/fimmu.2025.1673713

**Published:** 2025-09-29

**Authors:** Liang Dong, Zhi Cao, Weidong Han, Zhiqiang Wu

**Affiliations:** ^1^ Department of Bio-therapeutic, the First Medical Centre, Chinese People's Liberation Army (PLA) General Hospital, Beijing, China; ^2^ Changping Laboratory, Beijing, China; ^3^ National Clinical Research Centre for Hematologic Diseases, The First Affiliated Hospital of Soochow University, Suzhou, China

**Keywords:** glycosphingolipids (GSLs), sphingolipid metabolism, GSL storage disorders, cancer, therapeutic targets

## Abstract

Glycosphingolipids (GSLs) constitute the most structurally diverse subgroup of the sphingolipid family and play crucial roles in a wide variety of cellular functions. The expression of GSLs is tightly controlled during development, with each GSL series exhibiting distinct functional roles in adhesion or signaling, depending on cell type. Genetic defects in lysosomal GSL-degrading enzymes result in GSL storage disorders. However, aberrant and increased expression of GSLs has also been observed in various cancer cells, promoting tumor survival and impairing anti-tumor immunity. Additionally, viruses, pathogens, and bacterial toxins have been found to bind to host GSLs. Therefore, inhibiting GSL synthesis could be a potential therapeutic strategy for such infections or cancers. Here, we discuss the synthesis and classification of GSLs and review their role in disease and treatment.

## 1 Introduction

Cell membrane serves as both barriers and communication interfaces between distinct biological compartments. The surface of cellular membranes is densely populated with sphingolipids, among which GSLs represent the most structurally diverse subgroup (1). GSLs play critical roles in various cellular functions, and aberrant GSL expression has been observed in congenital diseases, infections, and cancer. However, due to their amphiphilic nature and inherent complexity, the biological significance, functional modifications of GSLs remain poorly understood (2–6). GSL subfamilies include the asialo-series, ganglio-series, globo-series, and (neo)lacto-series. These core structures can be further diversified through elongation, sulfation, sialylation, and other modifications. Overall, the number of unique GSL oligosaccharide structures exceeds 400 (7).

Aberrant GSL expression has been implicated in cancer cell transformation, metastasis, and multidrug resistance (8–10). Such as GM1, GM3 and GD2 play critical roles in tumor cell proliferation, migration, survival, and epithelial-mesenchymal transition (EMT), and can serve as prognostic markers and tumor-associated antigens for cancer progression (11–13). Thus, GSLs may function as diagnostic markers or therapeutic targets in cancer. Inherited deficiencies in enzymes acting downstream of glucosylceramide synthase (GCS) can lead to significant glycosphingolipid metabolic dysregulation, as seen in Gaucher disease (GD), Fabry disease (FD), Krabbe disease, and GM1/GM2 gangliosidosis (4). Enzyme replacement therapy (ERT) and substrate reduction therapy (SRT) are widely used in clinical practice to treat glycosphingolipid-related disorders (5).The association between GD and cancer has been frequently noted, with Gaucher patients exhibiting a higher incidence of B-cell malignancies (14, 15). In a Gaucher disease type 1 (GD1) mouse model, subcutaneous injection of melanoma cells resulted in accelerated tumor growth (16). These observations suggest that GD treatment strategies may influence the development and progression of associated cancers. Conversely, blocking tumor GSL synthesis could potentially activate the patient’s immune system, enabling targeted tumor destruction.

In this review, we focus on GSL synthesis, their physiological functions in mammals, and their role in disease. Finally, we discuss the potential of GSLs as therapeutic targets for cancer, particularly in the context of immunotherapeutic approaches in clinical practice.

## 2 Metabolic pathways and structures of GSLs

### 2.1 GSLs synthesis

**Figure 1:**
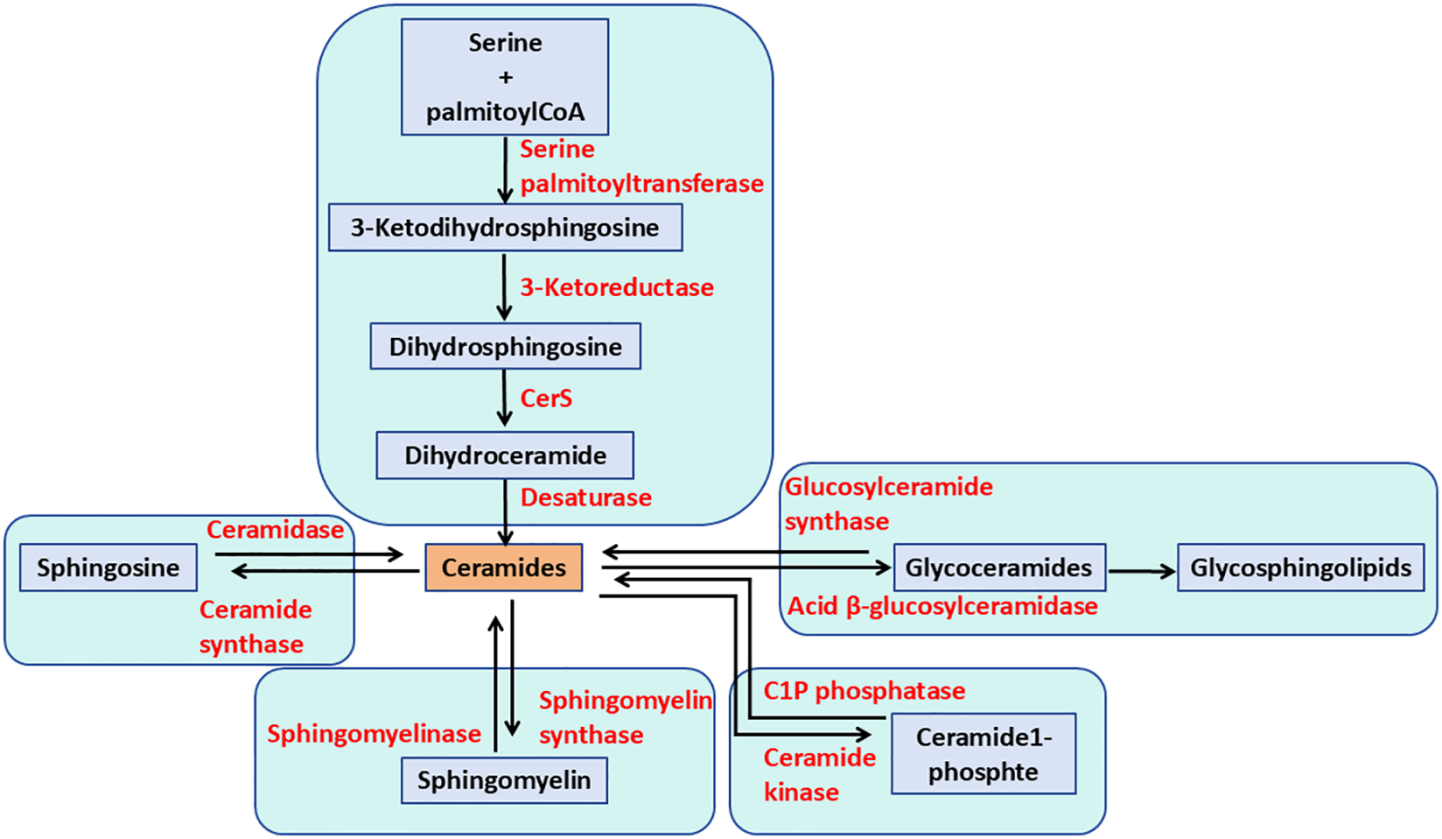
The pathways of ceramide metabolism. The *de novo* pathway of ceramide formation begins with the condensation of serine and palmitoyl-CoA, catalyzed by serine palmitoyltransferase. Ceramide can be phosphorylated to form ceramide-1-phosphate. Additionally, ceramide can be metabolized to sphingomyelin and glycosphingolipids through the actions of sphingomyelin synthases and glycosphingolipid synthases, respectively. Alternatively, ceramide can be hydrolyzed to sphingosine.

GSLs comprise a group of over 400 natural compounds derived from ceramides (Cer) and glycans. GSL metabolism begins in the endoplasmic reticulum (ER), where Cer is produced through the condensation of serine and palmitoyl-CoA, catalyzed by the serine palmitoyltransferase complex (SPT) (17). Six mammalian ceramide synthases (CerS1–CerS6) have been identified, each exhibiting distinct subcellular localizations. These enzymes are found in specific organelles, including the plasma membrane, lysosomes, mitochondria, the Golgi apparatus, and ER. In addition to GSLs, Cer can be converted into sphingomyelin (SM; phosphocholine-ceramide) or ceramide-1-phosphate (Cer1P), or degraded by ceramidases into fatty acids and sphingosine (18). CerS regulate both the *de novo* synthesis of sphingolipids and the recycling of sphingosine from the breakdown of pre-formed sphingolipids (**Figure 1**). Cer serves as an intermediate in the formation of various sphingolipids, which regulate multiple aspects of sphingolipid-mediated cell and organismal biology (19).

**Figure 2:**
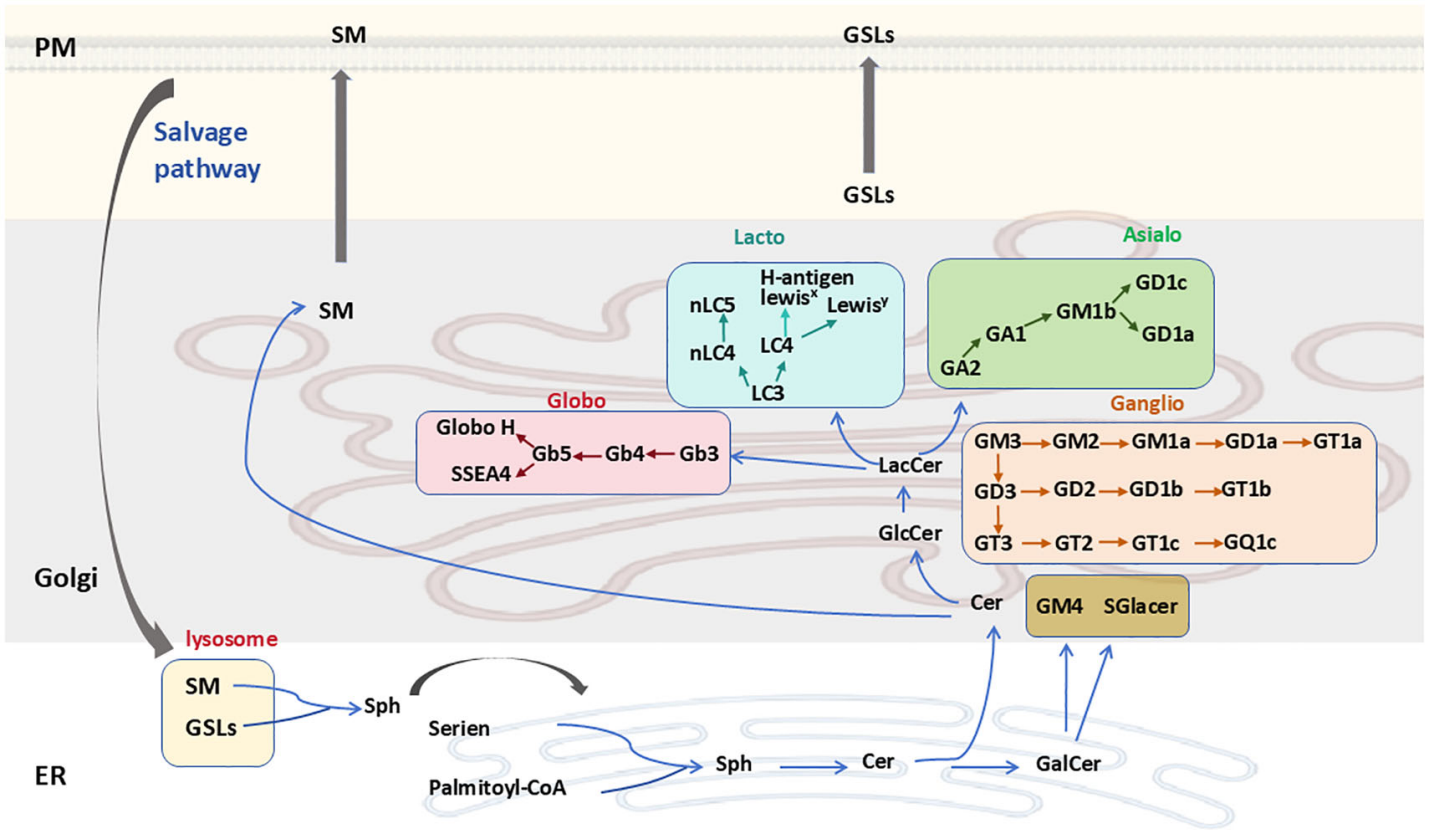
GSL synthesis and degradation. Cer is synthesized *de novo* in the ER and is then transported either via the ceramide transfer protein to the Golgi, where it serves as a substrate for the synthesis of SM or GlcCer. GSL biosynthetic pathways are marked with different colors. SM and GSLs are transported to the PM through vesicular trafficking and undergo vesicular trafficking in the endosomal system before being cleared via lysosomal degradation. Ceramide can also be converted to GalCer in the ER. GSLs, glycosphingolipids; Sph, sphinganine; SM, sphingomyelin; PM, plasma membrane; ER, endoplasmic reticulum.

In the ER, Cer can be galactosylated to produce galactosylceramide (GalCer), which is then transported to the Golgi complex, where it may undergo sulfation or sialylation (**Figure 2**). GalCer is the precursor of GSLs in the gala-series, catalyzed by the enzyme galactosylceramide synthase (CGT) using UDP-galactose as a sugar donor. GalCer is either sialylated to produce GM4 ganglioside or sulfated to produce sulfatide (2, 20). Alternatively, Cer can be transported via the ceramide-transfer protein (CERT) to the trans-Golgi network (TGN), where it is primarily used for sphingomyelin (SM) synthesis, which cannot undergo further anabolic processing. In another important pathway, Cer may reach the cis-Golgi, where the enzyme glucosylceramide synthase (GCS; encoded by the UDP-Glucose Ceramide Glucosyltransferase (UGCG) gene) converts Cer to GlcCer using UDP-glucose as a sugar donor (**Figure 2**) (7, 20).

Apart from the gala-series GSLs, all other GSLs have GlcCer as a precursor. GlcCer is translocated to the luminal leaflet of the Golgi, where TGN membranes convert it into lactosylceramide (LacCer; Gal-GlcCer). LacCer is synthesized by lactosylceramide synthase, encoded by the β4-galactosyltransferase genes 5 and 6 (B4galt5 and B4galt6), which catalyzes the transfer of galactose from UDP-galactose to GlcCer to form LacCer (21). LacCer represents the metabolic branch point for the formation of different classes of complex GSLs, which are classified into four categories: the globo, lacto, ganglio, and asialo series (**Figure 2**) (22). Specifically, LacCer serves as a substrate for β1,4-N-acetylgalactosylaminyltransferase (B4GALNT1) to produce GalNAcβ1-4Galβ1-4Glcβ1-Cer (GA2). It is also a substrate for α-2,3-sialyltransferase (ST3GAL5), yielding NeuAcα2-3Galβ1-4Glcβ1-Cer (GM3). The α1-4-galactosyltransferase (A4GALT) is responsible for producing Galα1-4Galβ1-4Glcβ1-Cer (Gb3), while β-1,3-N-acetylglucosaminyltransferase (B3GNT5) synthesizes GlcNAcβ1-3Galβ1-4Glcβ1-Cer (Lc3). Subsequently, GA2, GM3, Gb3, and Lc3 serve as precursors for the synthesis of GSLs belonging to the asialo, ganglio, globo, and lacto series, respectively (2, 7).

GSLs and sphingomyelin (SM) are transported to the outer plasma membrane via vesicular transport. Here, GSL composition can be further modified by plasma membrane-localized glycosidases (**Figure 2**). Additionally, GSLs are internalized from the plasma membrane into the endosomal/lysosomal system via endocytosis for degradation. Lysosomal GSL degradation is regulated by specific glycohydrolases, which facilitate the stepwise dismantling of glycan moieties, ultimately yielding less complex compounds (23, 24). The Cer within lysosomes is then catabolized by acid ceramidase, producing a fatty acid and sphingosine. The generated sphingosine is transported to the ER, where it is utilized for Cer synthesis via the salvage pathway (25).

### 2.2 Modification and complexity of GSLs

GSLs undergo extensive modifications in glycosylation pathways and serve as substrates for various reactions, leading to further metabolic diversification or the formation of branched glycan structures. Glycan elongation in GSLs is mediated by glycosyltransferases. The relative expression levels of glycosyltransferases, their subcellular localization, and multi-enzyme complex formation collectively determine the final GSL structure. Meanwhile, studies have shown that GSL polysaccharide synthesis follows strict structural rules (26). For instance, glucose (Glc) is always linked to galactose (Gal), fucose (Fuc) functions as a glycan chain terminator, sialic acid residues are elongated exclusively by other sialic acids, and Gal-GlcNAc repeats are highly prevalent (7). Additionally, sulfation of glycosphingolipids by sulfotransferase adds sulfate groups to the hydroxyl moieties of GSLs, further increasing their diversity (2). These observations suggest that GSL structural complexity results from a coordinated interplay between substrates and enzymes.

The ceramide backbone represents a second major contributor to GSL structural complexity (27). Currently, estimates suggest that 28 distinct enzymes are known to act on ceramide, either as substrates or products, for example, there are six ceramide synthases (CerSs), five ceramidases, and at least four sphingomyelinases (SMases) (28). Ceramide synthesis is complex mode of regulation, whereby each of the six mammalian CerSs generates ceramides with distinct acyl chain lengths. CerS1 primarily utilizes C18-CoA; CerS4 incorporates C18- and C20-CoAs; CerS5 and CerS6 predominantly use C16-CoA; and CerS3 specializes in very long-chain acyl-CoAs (C26 and longer). CerS2 exhibits broader substrate flexibility but primarily utilizes C22–C24 acyl-CoAs (29–34). The vast diversity of ceramides arises from combinatorial synthesis, where specific enzyme combinations produce one or a few ceramide species. Each ceramide serves as the foundation for a distinct complex sphingolipid (28). Thus, ceramide metabolism acts as a hub in sphingolipid biology, serving as a precursor for ceramide phosphate, sphingomyelin, ceramide phosphoethanolamine, and the entire GSL family.

In summary, although GSL synthesis and modification are not yet fully understood, current knowledge of GSL structures allows preliminary insights into their functions and properties. Given the vast array of molecules involved, it is essential to elucidate how individual cells determine their GSL composition and how specific GSLs interact with and regulate proteins, glycans, and lipids.

## 3 Expression and function of GSL

The expression of GSLs is strictly regulated during development, with each GSL series exhibiting distinctive cell- or tissue-type specificity and playing different functional roles in cell type-specific adhesion or signaling (35–37). Numerous studies have shown that stage-specific changes in GSL expression occur during mouse embryogenesis. The pre-implantation phase is dominated by GSLs of the lacto and globo series. During gastrulation, the production of ganglio-series GSLs is induced in both neuronal and glial cell precursors (36). Finally, during organogenesis, ganglio-series GSLs are the most abundantly synthesized. The relative amounts of gangliosides in the nervous system change dynamically from post-gastrulation (embryonic day 8, E8) to adulthood. At E12–E14, GD3 was the predominant ganglioside. After E16, the expression of GD3 and GM3 decreased markedly, while the expression of a-series gangliosides, such as GD1a, increased (38). These findings suggest that developmental processes involve the reprogramming of GSL metabolism.

The physiological role of GSLs has been studied using genetic, biochemical, biophysical, and cell biological approaches. Global deletion of the UGCG gene in mice, which eliminates all glucosylceramide (GlcCer)-based GSLs, causes early embryonic lethality (39). Similarly, ablation of the B4GALT-5 gene also leads to embryonic lethality. Ablation of B3GNT5 results in either preimplantation lethality or multiple postnatal defects (40). In contrast, ablation of the ST3GAL5 gene results in impaired neuropsychological behavior and hearing loss (41). Disruption of the CGT (ceramide galactosyltransferase) gene, which leads to the loss of all gala-series GSLs, induces profound neuronal phenotypes that appear secondary to defects in myelination (42). Collectively, this evidence suggests that the GSL composition of cells is reshaped during differentiation and that GSL synthesis plays a critical role in development.

## 4 Relationship between GSLs and disease

### 4.1 Lysosomal GSL storage disorders and therapies

GSLs are important building blocks of the cell membrane. They are continuously recycled, a process involving fragmentation within lysosomes by glycosidases. A number of human genetic metabolic disorders result from defects in the lysosomal enzymes involved in GSL degradation and are commonly referred to as “GSL storage disorder” (43). The common feature of GSL storage disorders is that the substrates of the defective enzymes accumulate in the lysosomes; the inability to degrade these compounds leads to a metabolic imbalance and the secondary accumulation of GSLs.

In Gaucher disease, for example, the enzyme glucosylceramidase beta 1(GBA1), which is required for the breakdown of GlcCer in lysosomes, is deficient. The main features of Gaucher disease are large “Gaucher cells”—macrophages with accumulated GlcCer in lysosomes—which concentrate in the spleen and bone marrow and are associated with neuronal abnormalities. The formation of splenic Gaucher cells is enhanced by rapid splenic clearance of defective red blood cells by macrophages (5). In Fabry disease (α-galactosidase deficiency), Krabbe disease (galactocerebrosidase deficiency), GM2 gangliosidosis (β-hexosaminidase deficiency), and Niemann-Pick disease types A and B (acid sphingomyelinase deficiency), the corresponding sphingoid bases of the accumulating substrates (lysoGb3, lysoGM2, and lysoSM, i.e. lysosphingolipids (LysoSLs):the N-deacylated forms of sphingolipids.) are formed, and their plasma levels are markedly elevated (43, 44). Although clinically distinct, sphingolipid disorders share some biochemical similarities. The effects of accumulated GSLs are thought to play an important role in the pathogenesis of these diseases. Patients with Gaucher disease are treated with either enzyme replacement therapy (ERT) or substrate reduction therapy (SRT), the latter involving the administration of UGCG inhibitors such as miglustat and eliglustat (5).

### 4.2 Infection

Studies have shown that viruses, pathogens, and bacterial toxins can bind to host GSLs and that the binding is necessary to induce pathological changes (45, 46). Several infectious pathogens and toxins are known to use GSLs as cellular receptors. A well-studied example is the human immunodeficiency virus (HIV). HIV entry into its host cells requires fusion of the viral envelope with the host cell membrane. Several GSLs have been identified as HIV-1 fusion receptors that are recognized by HIV gp120. These glycolipids include galactosylceramide (GalCer), 3′-sulfogalactosylceramide (SGC), monosialoganglioside (GM3), and globotriaosylceramide (Gb3) (47, 48).

Severe acute respiratory syndrome coronavirus-2 (SARS-CoV-2) is a novel virus with higher transmissibility (49). Host cellular Angiotensin-Converting Enzyme 2 (ACE2) serves as the viral receptor and mediates the process of SARS-CoV- 2 infection in human cells. While ACE2 plays a central role in virus-host interactions, other host cell surface molecules, such as gangliosides, have been proposed as potential co-receptors or attachment factors for ACE2-dependent SARS-CoV-2 entry (50, 51). Studies suggest that SARS-CoV-2 may achieve efficient cell entry through dual or even triple binding to ACE2 receptors and gangliosides on lipid rafts, forming a trimolecular complex. Recent molecular dynamics simulations of SARS-CoV-2 spike (S) protein interactions with model ganglioside GM1—a glycosphingolipid containing a single sialic acid residue—demonstrated that the glycan-binding domain (GBD) of the S protein forms a trimolecular complex with two GM1 molecules (52). These findings propose that SARS-CoV-2 S protein may bind to ganglioside-rich regions on the cell membrane, thereby promoting subsequent interactions between the receptor-binding domain (RBD) and ACE2.

Macauley et al. revealed that the RBD of SARS-CoV-2 S protein recognizes monosialylated gangliosides (53). Using catch-and-release electrospray ionization mass spectrometry (CaR-ESI-MS) screening of glycan libraries, they identified the pentasaccharide of ganglioside GM1 as the RBD’s preferred ligand. Subsequent experiments with artificial membranes embedded with gangliosides confirmed RBD specificity, showing that GM1, GM2, and GM3 gangliosides were all recognized by the RBD. Einat B. Vitner et al. reported that two GCS inhibitors Genz-123346 and GENZ-667161 inhibit the early stages of SARS-CoV-2 replication. However, the precise mechanism by which GCS inhibitors block viral replication remains unclear (54). In summary, inhibition of the sphingolipid synthesis pathway may represent a potential therapeutic target for multiple viral infections.

The polyomavirus invades human erythrocytes via the gangliosides GD1a and GT1b. GM1 has also been shown to act as a receptor for simian virus 40 (SV40) and polyomavirus (55). Meanwhile, infection with different pathogens can lead to changes in the composition of the cell surface GSL repertoire. Cytomegalovirus (CMV) induces increased synthesis of (neo)lactoseries GSLs (56). The p40tax protein encoded by the human T-cell lymphotropic virus can induce GD2 expression by upregulating B4GALNT1 (57). A potential reason for such dysregulation may be to evade detection and elimination by the immune system.

Several infectious pathogens and toxins use GSLs as cellular receptors. A variety of bacterial toxins target GSLs via their binding subunits (B subunits) to deliver enzymatically active subunits (A subunits) into host cells. Cholera toxin, one of the most well-characterized toxins, has been demonstrated to specifically bind GM1 (58). Similarly, enterotoxin B was historically considered specific for GM1 (59). The B subunits of Shiga toxins (STx) and verotoxins bind Gb3 and induce endocytosis (60, 61). Studies have reported that gangliosides (such as GM1) on activated CD4+ T cells interact with the O-antigen polysaccharide moiety of lipopolysaccharide (LPS)—the major surface antigen of Shigella—thereby promoting bacterial adhesion to these T cells (62). Furthermore, many bacteria possess the ability to bind GSLs, although the underlying pathophysiological significance of this phenomenon remains unclear. For example, Helicobacter pylori can bind to sialic acid-containing GSLs on neutrophils (63). The ganglioside asialo-GM1 on the surface of epithelial cells binds Bifidobacterium bifidum, Pseudomonas aeruginosa, and Lactobacillus (64). Ganglioside GM1 has been implicated in infections with Brucella species (65). Fimbriated E. coli bind to the globo series Gb3 and Gb4 (66). Virulent strains of Bordetella pertussis, a human respiratory pathogen, bind with high affinity to sulfatide (67). The neutral GSL LacCer at the surface of intestinal epithelial cells binds various microorganisms, including Candida albicans, B. pertussis, Mycobacterium tuberculosis, E. coli, Bacillus dysenteriae, and Propionibacterium freudenreichii (68, 69). There is evidence that the adhesion of Helicobacter pylori—which causes chronic active gastritis, peptic ulcer disease, and gastric adenocarcinoma—depends on gangliosides in the human stomach. Despite the health risks associated with GSL expression, specific GSLs play an important role in physiological functions, including their multiple roles in immunity.

### 4.3 Cancer

Aberrations in GSL metabolism have also been linked to cancer. In fact, cells rearrange their GSL composition during oncogenic transformation, with characteristics similar to those observed in normal embryonic development and tissue lineage differentiation processes (70, 71). This rearrangement has been suggested to contribute to phenomena such as cell-cell adhesion, cell-matrix interaction, epithelial-to-mesenchymal transition (EMT), tumor proliferation, invasion, metastasis, angiogenesis, and the emergence of multidrug resistance (10, 72). The high levels of GSLs in tumors can interact with antigen-presenting cells through their binding to glycan-binding receptors, thereby inducing immunosuppressive signals and impairing the killing capacity of the immune system (73). GSLs can serve as a source for the development of novel clinical biomarkers, providing a set of specific targets for therapeutic intervention (74).

GSL reprogramming plays a role in EMT, the key process that enables metastatic cell invasion during cancer progression. During EMT, the production of GSLs shifts from the asialo to the ganglio series due to the induction of ST3GAL5 and ST8SIA1 (encoding GD3 synthase) and the repression of B3GALT4 (encoding GA1/GM1 synthase) (75). Induction of EMT *in vitro* by transforming growth factor β (TGFβ) treatment is accompanied by a reduction in the levels of asialo-GSLs GM1 and GM2, while the synthesis of complex gangliosides with promoters of both ST3GAL5 and ST8SIA1 is induced during this process (7, 76). Research has shown that the loss of globo series due to deletion of A4GALT in cells results in EMT, whereas deletion of ST8SIA1 induces epithelial cell characteristics. Studies demonstrate that a subpopulation of Stage Specific Embryonic Antigen-4 (SSEA-4) positive prostate cancer cells forms fibroblast-like colonies, accompanied by downregulation of epithelial cell-associated markers such as Claudin-7, E-cadherin, Epithelial Splicing Regulatory Protein 1(ESRP1), and Grainyhead-like 2(GRHL2), whereas SSEA-4 negative cells form cobblestone-like epithelial colonies (77). These findings suggest that targeting GSL synthases may be a novel approach to prevent cancer recurrence.

Indeed, numerous studies have shown that GSLs regulate cellular signaling pathways by interacting with components of the signal transduction machinery (e.g., hormones, receptors, and intracellular transducers). Clusters of GSLs on the cell surface membrane interact with functional membrane proteins such as growth factor receptors. A classic example is the interaction between the epidermal growth factor receptor (EGFR) and GM3. EGFR activation or inactivation depends on the GSL composition of the membrane in which it resides (78). Studies have found that exogenously added GM3 inhibits cell growth in different cell lines by modulating EGF receptor phosphorylation. Inhibition of EGF receptor autophosphorylation by GM3 in liposomes depends on the presence of the NeuAc residue in GM3 and lysine 642 in the EGF receptor. Through this interaction, GM3 maintains EGFR in its resting state, preventing receptor dimerization and activation (79, 80).

The globo series GSL Gb3 regulates the receptor function of Fas (CD95). Fas has a GSL-sensing domain (GSD) that interacts specifically with Gb3 and LacCer but not with Gb4 or gangliosides. The Fas-GSL interaction has important functional consequences, as the GSD of Fas determines its internalization route—a key mechanism for eliminating pathogen-infected cells and controlling autoimmune diseases and certain malignancies (81).

Tumors often exhibit high levels of GSLs, which interfere with the cytotoxic efficacy of the immune system. These elevated GSL levels lead to significant concentrations of free GSLs within the tumor microenvironment, either through active or passive mechanisms. In patients with neuroblastoma, the plasma concentration of tumor-derived GSLs was found to be 50 times higher compared to post-treatment levels or healthy controls (82). Multiple mechanisms have been proposed for the immunosuppressive effects of free GSLs. Research has shown that tumors shed gangliosides, which block the proliferative response of T cells by directly binding to a lectin-like site on IL-2, thereby inhibiting IL-2 binding to its receptors on T cells (83). T cells isolated from renal cell carcinoma were found to be GM2-positive and exhibited higher apoptosis rates compared to their GM2-negative counterparts. This suggests that T cells with minimal GM2 synthase mRNA expression acquire GM2 from the tumor microenvironment (84).

Additionally, CD4+ T cells cultured in the presence of GT1b shifted from an IFN-γ-secreting type-1 phenotype to an IL-4-producing type-2 phenotype (85). *In vivo* mouse models indicate that cytotoxic CD8+ T cell populations are also affected by ganglioside exposure, in terms of expansion and tumor-specific responses to secondary challenges with tumor cells. Furthermore, gangliosides prevent T-cell receptor (TCR)-induced lytic granule polarization and immunological synapse accumulation (86). Similarly, LPS-stimulated monocytes preincubated with GD1a showed inhibited CD80 upregulation, decreased CD40 levels, and impaired release of IL-12 and TNF-alpha (87). This impaired response of activated dendritic cells (DCs) is also observed with GM3 and GD3. Moreover, pre-incubation of monocytes with GM2 and GM3 impairs Fc receptor expression, reduces IL-1 production, and decreases Toll-like Receptor (TLR) signaling (88). Importantly, the number and function of tumor-infiltrating myeloid-derived suppressor cells (MDSCs) were significantly reduced in ganglioside-deficient tumors. Transient ganglioside reconstitution in ganglioside-deficient tumors was sufficient to increase MDSC infiltration, favoring immune escape (89). Similarly, GM2 and GM3 were found to be potent inhibitors of NK cell activity, as tumor gangliosides bind to inhibitory receptors such as Siglec-7 and -9 (90–92). In summary, high concentrations of gangliosides shed by tumors lead to a downregulation of the cellular immune response.

## 5 Targeting GSL in the treatment for disease

### 5.1 Treatment for GD

Enzyme Replacement Therapy (ERT) is a successful therapeutic intervention for type 1 Gaucher disease (GD), in which the enzyme deficiency in the patient’s macrophages is supplemented by repeated intravenous infusions of therapeutic recombinant glucocerebrosidase (GCase). To enhance GCase targeting of macrophages, the enzyme is modified with N-linked glyco-ligands containing terminal mannose groups to favor uptake via the mannose receptor(CD206)present at the surface of tissue macrophages (Mannose receptor is primarily expressed by macrophages, dendritic cells and endothelial cells and is involved in scavenging events.) (93, 94). Biweekly ERT in type 1 GD patients dramatically reverses visceral symptoms, such as hepatosplenomegaly, and corrects hematological abnormalities. Unfortunately, ERT does not prevent neurological symptoms because the enzyme cannot cross the blood-brain barrier (BBB) (5).

Substrate Reduction Therapy (SRT) is an approved alternative treatment for type 1 GD. It aims to balance the synthesis of GlcCer with the impaired ability of GD patients to break it down. Currently, two oral inhibitors Miglustat and Eliglustat are approved for treating type 1 GD patients (95, 96). Miglustat is a relatively weak inhibitor of glucosylceramide synthase (GCS) (with an IC50 in the micromolar range) and also inhibits off-target intestinal glycosidases, particularly non-lysosomal GBA2 (with an IC50 in the nanomolar range) (97, 98). Although it is brain-permeable, it is not currently approved for treating neuronopathic GD (99). In contrast, Eliglustat, a more potent and specific inhibitor, has been shown to produce visceral improvements in patients comparable to ERT. However, Eliglustat does not effectively cross the BBB, making it unsuitable for treating central nervous system (CNS) manifestations in neuronopathic GD (types 2 and 3), GM2 gangliosidosis, or GBA-associated Parkinson’s disease (GBA-PD) (100, 101). A novel brain-penetrant GCS inhibitor, Venglustat (ibiglustat, GZ/SAR402671), is being developed by Sanofi Genzyme for the treatment of Fabry disease, Parkinson’s disease, type 3 GD, and GM2 gangliosidosis (102). Venglustat has been evaluated for safety and efficacy in Fabry disease in a Phase 2 clinical trial (NCT02228460) and in Gaucher disease type 3 in the Phase 2 open-label LEAP trial (NCT02843035) (103, 104).

Another promising inhibitor, AMP-DNM [N-(5-adamantane-1-yl-methoxypentyl)-deoxynojirimycin], is an iminosugar-based GCS inhibitor. These orally available nanomolar-range GCS inhibitors can modulate glycosphingolipid (GSL) metabolism in the brains of mice and have been shown to improve disease outcomes in models of Niemann-Pick disease and Sandhoff disease (105, 106). Thus, inhibiting GCS or reducing upstream GSL metabolites (such as globo series GSLs and gangliosides) holds therapeutic potential for lysosomal storage diseases, including GD, Fabry disease, GM2 gangliosidosis (Tay-Sachs disease, Sandhoff disease), GM1 gangliosidosis, and Niemann-Pick disease.

### 5.2 Treatment for cancer

**Figure 3:**
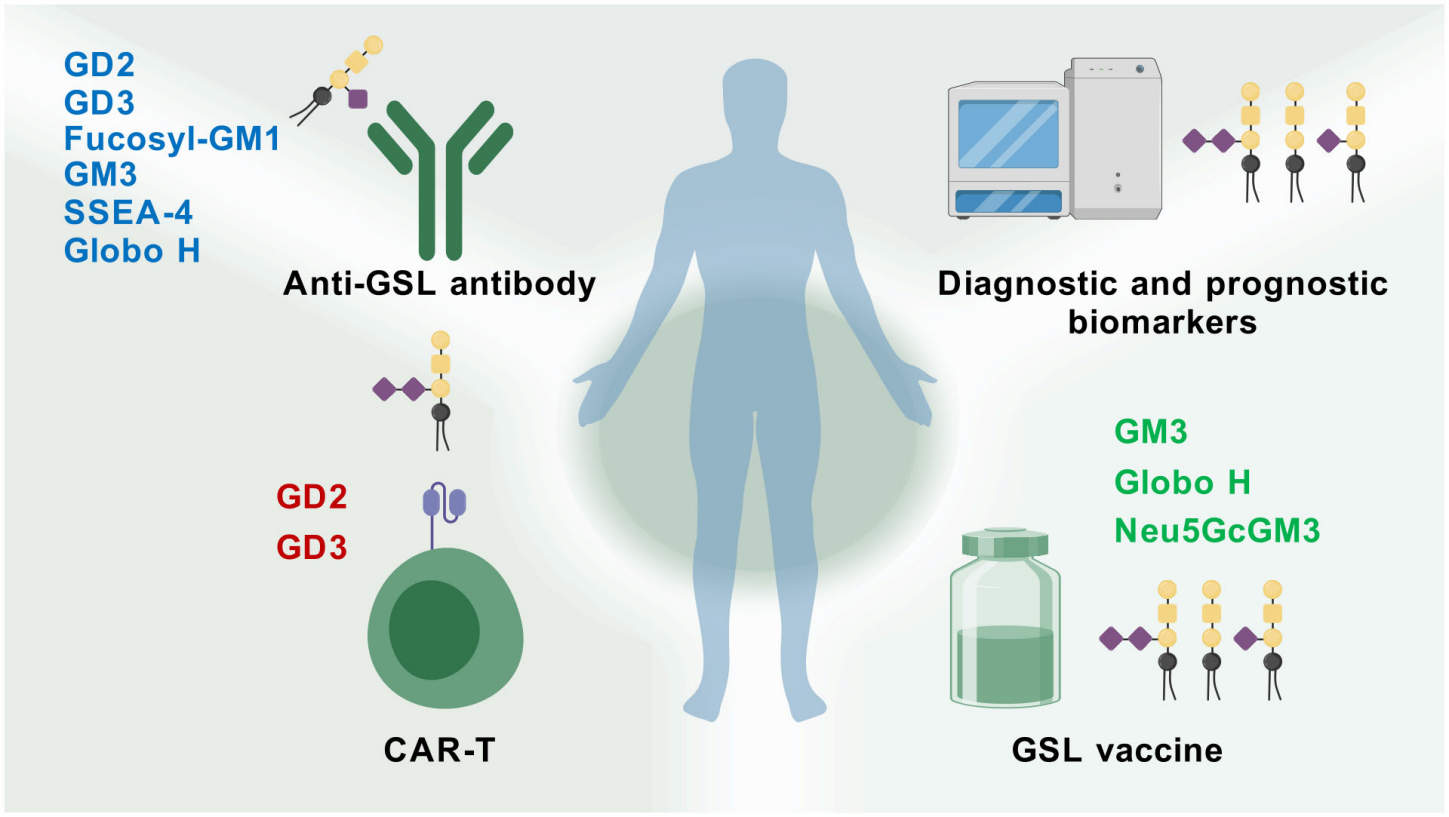
Therapy potential of GSLs for cancer. GSL-related vaccines, antibodies, CAR-T therapies, and glycolipid microarray analysis have been developed and applied in cancer research (Created with BioGDP.com).

GSLs actively modulate various roles in cellular biology, including apoptosis, cell proliferation, endocytosis, intracellular trafficking, cell migration, senescence, and inflammation (19, 107). The occurrence of tumors has been associated with the overproduction of specific GSLs, which are critical factors in tumorigenesis, cancer progression, and the efficacy of anti-cancer therapies (108). Inhibiting the synthesis of tumor-associated GSLs may enable patients to antigen exposure and activate T cells that can destroy tumors (109). Tumors exhibiting multidrug resistance may do so by synthesizing GSLs even faster than usual (110). Inhibiting their synthesis of GSLs could restore the tumor’s sensitivity to anti-cancer drugs. Metastasis of tumors also appears to require GSLs, so an inhibitor could help block tumor dissemination. Moreover, a large number of tumor-associated antigens have been identified as GSLs (111). For more than twenty years, these tumor-associated carbohydrate antigens (TACAs) have demonstrated potential usefulness in defining tumor type and stage. Importantly, GSLs that serve as tumor-associated antigens (TAAs) have been targeted through approaches such as active immunity induced by vaccines, monoclonal antibodies developed by genetic engineering, bispecific antibodies, and chimeric antigen receptor-T (CAR-T) cells (**Figure 3**).

To categorize and identify tumor-associated antigens, the National Cancer Institute of the United States conducted a comparative analysis based on established objective criteria, including potential therapeutic efficacy, expression levels, immunogenicity, and the percentage of positive cells. The study showed that among the top 75 cancer antigens, four were GSLs: GD2 (disialoganglioside, GalNAcβ1-4NeuAcα2-8NeuAcα2-3Galβ1-4Glcβ1-1-Cer]), GD3 (disialoganglioside, NeuAcα2-8NeuAcα2-3Galβ1-4Glcβ1-1-Cer), GM2 (monosialoganglioside, GalNAcβ1-4Neu5Acα2-3Galβ1-4Glcβ1-1-Cer), and GM3 (monosialoganglioside, Neu5Acα2-3Galβ1-4Glcβ1-1-Cer) (111). GD2, a type of ganglioside, has three approved anti-GD2 drugs—dinutuximab (Unituxin^®^), dinutuximab-beta (Qarziba^®^), and naxitamab (Danyelza^®^)—used in clinical practice for treating high-risk neuroblastoma (112). These anti-GD2 mAbs include murine mAbs (3F8, 14.18, ME36.1), chimeric mAbs (ch14.18), and humanized mAbs (hu14.18, hu3F8). Clinical trials of anti-GD2 therapies have been conducted in patients with neuroblastoma, breast cancer, osteosarcoma, and leiomyosarcoma (NCT05489887, NCT06026657, NCT02502786, NCT05080790, etc.) (113). GD3, another disialic ganglioside, is synthesized during development and in cancers of neuroectodermal origin. An anti-GD3 antibody-drug conjugate (PF-06688992), composed of humanized anti-GD3 huR24 linked to a chemotherapeutic agent, was tested in a Phase I clinical trial for stage III or IV melanoma (NCT03159117) (114). Fucosyl-GM1 (FucGM1) is expressed in human small cell lung cancer (SCLC) and is being targeted by the antibody BMS-986012, currently in a phase I/II clinical trial as first-line therapy for extensive-stage SCLC (NCT02815592). A phase I/II study evaluating BMS-986012 alone and in combination with nivolumab in relapsed/refractory SCLC (NCT02247349) demonstrated that BMS-986012 is well tolerated and shows antitumor activity in some patients (115–117). Ganglioside GM3 is widely distributed in animal cells and overexpressed in melanomas, lung cancer, and brain cancer. A GM3 antibody is also undergoing preclinical investigation by Morphotek.

Another option is vaccinating with GSLs or structures bearing GSL antigens to induce an antibody response against GSLs overexpressed by a patient’s tumor. TACAs are shared by various cancer cell types, including Lewis y and ganglioside GD2 in breast cancer and GM2, GD2, and GD3 gangliosides in brain tumors. As such, vaccines against TACAs could target multiple cancer types (118). However, as a “self” antigen, GD2 is poorly immunogenic, making it difficult to induce a specific anti-GD2 immune response *in vivo* (119). Therefore, vaccines have been developed that use pseudo-glycoproteins generated by attaching glycans to KLH as haptens, rather than using GSLs directly. A bivalent vaccine containing GD2-GD3-KLH/QS-21 was evaluated in a phase I trial for high-risk neuroblastoma patients. A trivalent vaccine (GM2-GD2-GD3-KLH/QS-21), with KLH as a carrier protein and QS-21 or OPT-821 as adjuvants, was tested in metastatic sarcoma patients (NCT01141491) (120). A phase III clinical trial (EORTC18961) in 970 stage II melanoma patients receiving GM2-KLH/QS-21 vaccinations showed prognostic serum antibody responses correlated with favorable outcomes (121).

A hexavalent vaccine was evaluated in a phase II trial involving 30 high-risk prostate cancer patients. The vaccine included GM2, Globo H, Lewis y, glycosylated MUC-1-32mer, Tn, and TF in a clustered formation, conjugated to KLH and mixed with QS-21. All 30 patients had significant elevations in antibody titers to at least two of the six antigens (122). The anti-idiotype vaccine Racotumomab (Vaxira^®^) mimics the ganglioside GM3 (Neu5Gc). A phase I trial in pediatric neuroectodermal malignancies (NCT01598454) confirmed that Vaxira^®^ has a favorable toxicity profile at doses up to 0.4 mg, with most patients eliciting an immune response (123). Anti-idiotype vaccines are designed based on Jerne’s idiotypic network theory. According to this theory, upon exposure to an antigen, the host immune system first produces antibodies against the antigen (Ab1), followed by antibodies targeting the variable region of Ab1 (known as anti-idiotype antibodies, Ab2), then antibodies against the variable region of Ab2 (Ab3), and so on. Ab2 can be classified into four types: Ab2α, Ab2β, Ab2γ, and Ab2δ. Among them, Ab2β can mimic the structure of the original antigen and thus serves as a surrogate antigen for the development of anti-idiotype vaccines (124). Racotumomab-alum (Vaxira^®^) has been approved in Latin American countries for advanced NSCLC treatment and can mediate an antigen-specific antibody dependent cell mediated cytotoxicity (ADCC) response in NSCLC patients (RPCEC00000009) (125). Thus, targeting tumor-expressed GSL TACAs likely represents the most effective anticancer strategy.

CAR-T cells combine antibody specificity with the lytic capacity of T cells in an MHC-independent manner (126). Some TACAs, particularly GD2 and GD3, have proven useful for anti-tumor CAR-T engineering. The first clinical trial using first-generation GD2-targeting CAR-T cells recruited 11 neuroblastoma patients, demonstrating safety and showing tumor necrosis or regression in 4 of 8 evaluable patients. A follow-up study with 19 patients (including the original 11) reported that CAR-T cell persistence correlated with longer progression-free survival, with 3 of 11 achieving complete remission (127). A phase I trial of 4th‐generation CAR-GD2 T cells in 12 children with relapsed/refractory neuroblastoma (NCT02765243) showed stable disease in 6 of 10 patients at 6 months, with 4 remaining stable at 1 year and alive after 3–4 years of follow-up, without neurotoxicity (128). In a phase 1–2 trial, third-generation GD2-CAR T cells treated 27 children with relapsed/refractory high-risk neuroblastoma, yielding a 63% overall response rate (9 complete, 8 partial responses) (129). Beyond neuroblastoma, GD2-targeting CAR-T cells have shown antitumor activity in melanoma xenograft models (130). GD3 as a CAR-T antigen has also been explored preclinically, with a 50% complete response rate in mice treated with second-generation anti-GD3 CAR-T cells plus IL-2 (131).

Currently, two specific treatments exist for GD: ERT and SRT. In cancer patients, tumors secrete excessive GSLs, blocking immune-mediated tumor attack. Inhibiting tumor GSL synthesis could enable patients to generate antibodies and activate the immune system to destroy tumors. Mouse models show that GSL synthesis inhibition reduces tumor burden or even cures the disease (132). Recently, eliglustat has gained attention for cancer treatment (133, 134). Multidrug-resistant tumors may overproduce GSLs, and inhibiting their synthesis could restore drug sensitivity (109). Additionally, high GSL expression impairs T cell and DC function, suggesting GSL synthesis inhibition could benefit cancer immunotherapy (86, 135). Studies have demonstrated an unusual preference of Siglec-7 for a2,8-disialylated structures over terminal a2,3- and a2,6-linked sialic acids (92, 136–138). Theruvath J et al. studies show that blocking the GD2-Siglec7 axis increases M1 macrophages and reduces M2 tumor associated macrophages (TAM) polarization (139). GSL interactions with Siglecs contribute to the immunosuppressive tumor microenvironment; for example, high GSL levels interfere with HLA-I binding to immune receptors, impairing CD8+ T cell activation. Eliglustat or miglustat-mediated GSL synthesis inhibition enhances antitumor immunity *in vitro* (109). A phase I trial of a GSL synthase inhibitor has been conducted in advanced relapsed/refractory hematological malignancies and solid tumors (134).

## 6 Conclusions and perspectives

GSLs are amphiphilic molecules that comprise a vast group of biological polymers, showing remarkable heterogeneity in their structures. Meanwhile, GSLs are specifically expressed in mammalian cell membranes under certain developmental and pathological conditions (7). Thus, specialized GSLs have important biological functions in extracellular and intercellular signaling pathways. These GSLs not only affect cell phenotype at the nongenetic level but also shape cell and organism phenotype at the epigenetic level (140). Therefore, GSLs are closely associated with human diseases, such as neurodegenerative diseases, autoimmune diseases, metabolic diseases, and cancer. Analysis of cancer-associated GSLs and their metabolic enzymes is important for a deeper understanding of the physiological functions of GSLs.

Many studies have shown that GSLs have broad application prospects in tumor diagnosis and treatment. Combined with the differential expression and pathological characteristics of GSLs, they can be used to predict drug sensitivity, tumor metastasis, and recurrence. Thus, GSLs have great potential as diagnostic and prognostic biomarkers. Research on GSL-related vaccines, antibodies, and CAR-T cells is growing, which could inspire more important cancer immunotherapy strategies. The synergy of these GSL-related molecules with other anti-cancer drugs may maximize therapeutic efficacy and provide more diverse options for individualized therapy. However, compared to other molecules, little is known about the regulatory targets, expression patterns, and structural and functional roles of GSLs. The main reason is the technical challenges, resulting in unclear structural and functional features of GSLs. Thus, determining the GSL composition of a biological sample remains an analytical challenge.

The composition and expression of GSLs vary significantly in abundance, chemical stability, and biophysical properties, making their uniform extraction from biological samples difficult. In addition, heterogeneous localization and sugar chain branching further complicate GSL analysis. However, the accuracy in resolving GSL composition has improved with advancing technologies. The development of MS-based optochemical strategies for cross-linking GSLs has provided a fast and reliable method for determining GSL levels and structures in biological samples (141). Technological advances may have important implications for understanding the molecular mechanisms and developing therapeutic strategies for cancer immunotherapy by targeting GSLs.
